# Supplementary material for: Causal Relationship between Aging and Anorexia Nervosa: A White-Matter-Microstructure-Mediated Mendelian Randomization Analysis
Source: Biomedicines. 2024 Aug 16;12(8):1874. doi: 10.3390/biomedicines12081874 (PMC11351342; doi:10.3390/biomedicines12081874)
Supplement: Supplementary file 1 [file biomedicines-12-01874-s001.zip › Supplementary Figure/Figure S8/Leaveoneout plot of MR analysis for TL to ubm-b-2050.pdf]

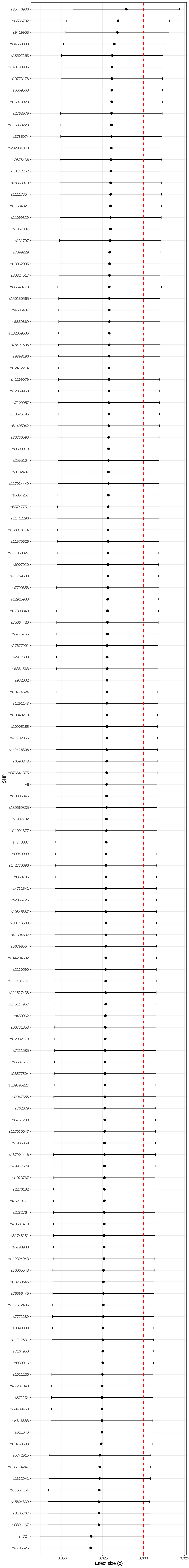
rs35446936
rs6536702
rs9419958
rs34550383
rs28502153
rs143190905
rs10773176
rs6669563
rs16978028
rs2763979
rs116863223
rs3785074
rs202034370
rs9878436
rs10112752
rs28363070
rs11117354
rs11584821
rs11699829
rs1957937
rs131797
rs7099229
rs13062095
rs80324517
rs35640778
rs150150565
rs4695407
rs6659669
rs182059586
rs78491606
rs9398196
rs12412214
rs41269079
rs12369950
rs7209057
rs113525195
rs61405042
rs73730598
rs9600019
rs2555104
rs8102497
rs117034449
rs6054257
rs55747751
rs11412296
rs188918174
rs11579626
rs111950327
rs6007020
rs11769630
rs7790856
rs12925933
rs17803849
rs75664430
rs6776756
rs17677991
rs2977608
rs6881568
rs932002
rs10774624
rs1291143
rs10840270
rs10905255
rs77732866
rs142426306
rs6590343
rs376641875
All
rs10805346
rs139669835
rs1907702
rs11991877
rs4743037
rs9940099
rs142730696
rs869785
rs4731541
rs2056726
rs10845387
rs80116508
rs41304832
rs56799554
rs144204502
rs2230590
rs117407747
rs111527438
rs145114957
rs450962
rs66731853
rs12932179
rs7221585
rs6587577
rs28577594
rs139795227
rs2967355
rs762679
rs6751209
rs117630647
rs1985369
rs137901416
rs79977579
rs1023767
rs2276182
rs76219171
rs2282764
rs73581419
rs61748181
rs6790988
rs112394943
rs76065543
rs13230646
rs76666449
rs117512405
rs7772289
rs3093888
rs11212631
rs7164950
rs939916
rs1611236
rs77231040
rs871134
rs59409453
rs4616688
rs611646
rs10768683
rs5742915
rs185174247
rs1332941
rs11557154
rs45604339
rs8105767
rs3891167
rs4724
rs7705526
SNP
−0.050
−0.025
0.000
0.025
Effect size (b)
